# FDG-PET hypermetabolism is associated with higher tau-PET in mild cognitive impairment at low amyloid-PET levels

**DOI:** 10.1186/s13195-020-00702-6

**Published:** 2020-10-19

**Authors:** Anna Rubinski, Nicolai Franzmeier, Julia Neitzel, Michael Ewers, the Alzheimer’s Disease Neuroimaging Initiative (ADNI)

**Affiliations:** 1 Institute for Stroke and Dementia Research, Klinikum der Universität München, Ludwig-Maximilians-Universität LMU, Feodor-Lynen-Straße 17, 81377 Munich, Germany; 2 German Center for Neurodegenerative Diseases, Munich, Germany

**Keywords:** FDG-PET, Hypermetabolism, Tau-PET, Amyloid-PET, Hyperactivation, Mild cognitive impairment

## Abstract

**Background:**

FDG-PET hypermetabolism can be observed in mild cognitive impairment (MCI), but the link to primary pathologies of Alzheimer’s diseases (AD) including amyloid and tau is unclear.

**Methods:**

Using voxel-based regression, we assessed local interactions between amyloid- and tau-PET on spatially matched FDG-PET in 72 MCI patients. Control groups included cerebrospinal fluid biomarker characterized cognitively normal (CN, *n* = 70) and AD dementia subjects (*n* = 95).

**Results:**

In MCI, significant amyloid-PET by tau-PET interactions were found in frontal, lateral temporal, and posterior parietal regions, where higher local tau-PET was associated with higher spatially corresponding FDG-PET at low levels of local amyloid-PET. FDG-PET in brain regions with a significant local amyloid- by tau-PET interaction was higher compared to that in CN and AD dementia and associated with lower episodic memory.

**Conclusion:**

Higher tau-PET in the presence of low amyloid-PET is associated with abnormally increased glucose metabolism that is accompanied by episodic memory impairment.

## Introduction

In Alzheimer’s disease (AD), alterations in glucose metabolism as assessed by [^18^F]fluorodeoxyglucose positron emission tomography (FDG-PET) are a common pathological hallmark [1]. Specifically, FDG-PET hypometabolism within temporoparietal regions is commonly observed in AD dementia and earlier AD stages, including in amyloid-positive mild cognitive impairment (MCI; i.e., prodromal AD) [2] and cognitively normal (CN) elderly at genetic risk of AD [3]. However, FDG-PET metabolism shows complex changes during the course of AD, where not only reductions but also increases in FDG-PET metabolism have been reported across CN amyloid-positive subjects [4] and subjects at genetic risk of AD [5, 6] and MCI [7]. Thus, clinical staging of cognitive symptoms does not correspond to FDG-PET alterations in a straightforward manner.

Studies using amyloid- and tau-PET imaging suggest that these pathologies are important predictors of regional FDG-PET alterations. For amyloid-PET, elevated global levels of amyloid-PET have been associated with reduced FDG-PET in both AD dementia [8] and MCI [9]. However, increased FDG-PET has also been observed in association with elevated amyloid-PET [4]. Furthermore, there is a poor regional match between amyloid-PET and FDG-PET in typical [10] and atypical AD [11] suggesting that amyloid-PET alone cannot fully account for FDG-PET alterations. Results from tau-PET studies suggest that tau pathology may be an important modulating factor of FDG-PET [12–14]. Results from recent studies in elderly asymptomatic CN revealed an interaction between amyloid- and tau-PET, where higher tau-PET was associated with *higher* FDG-PET at low levels of amyloid-PET, but with lower levels of FDG-PET at high levels of amyloid-PET [15, 16]. These results provide an intriguing model of the dynamic bidirectional changes in relationship to beta-amyloid (Aβ) and tau pathology. The focus on biomarkers of Aβ and tau pathology rather than the clinical diagnosis of AD allows to investigate the effect of different mixtures of both pathologies on FDG-PET changes and cognitive impairment. This is important because even in the absence of abnormal levels of Aβ, abnormal tau-PET levels can be observed in higher cortical brain areas in a substantial number of elderly subjects, where higher tau-PET was associated with cognitive impairment [17]. However, the association of higher tau-PET with FDG-PET alterations at varying levels of Aβ in symptomatic elderly subjects is unclear. In order to address this research gap, we examined both the main and interaction effects of [^18^F]AV45 amyloid-PET and [^18^F]AV1451 tau-PET on FDG-PET in subjects with amnestic MCI. Furthermore, we tested whether the observed higher levels of FDG-PET represent abnormally increased FDG-PET, i.e., FDG-PET hypermetabolism, and whether such increases in FDG-PET are beneficial or detrimental for cognition.

## Methods

### Participants

All subjects were recruited within the Alzheimer’s Disease Neuroimaging Initiative (ADNI phase III; http://adni.loni.usc.edu/) [18]. Inclusion criteria for the current study beyond those of ADNI were a diagnosis of MCI at the PET acquisition visit (Mini-Mental State Examination (MMSE) > 24, Clinical Dementia Rating (CDR) = 0.5, objective memory loss on the education-adjusted Wechsler Memory Scale II, preserved activities of daily living) and the availability of [^18^F]AV1451 tau-PET, [^18^F]AV45 amyloid-PET, and [^18^F]FDG-PET up to 6 months apart. From the total sample of 74 MCI subjects fulfilling the inclusion criteria, two subjects failed preprocessing and were excluded, yielding a final sample of 72 MCI subjects. Apolipoprotein E (APOE) genotyping was available as well.

In addition to the MCI group with all three PET modalities, a group of 70 cerebrospinal fluid (CSF) Aβ- and p-tau_181_-negative CN subjects (MMSE > 24, CDR = 0) and 95 AD dementia subjects (MMSE < 26, CDR > 0.5, fulfillment of NINCDS/ADRDA criteria for probable AD) [19] were also included to assess group-level differences in regional FDG measures. These subjects were recruited in ADNI phase II and were selected for the current study based on the availability of FDG-PET and CSF biomarkers of Aβ and tau. CN subjects were asymptomatic and Aβ and phosphorylated tau (p-tau) negative based on a quantitative CSF threshold (Elecsys CSF immunoassay; Aβ_1–42_ > 976.6 pg/ml, p-tau_181_ < 21.8 pg/ml [20];). AD dementia subjects were diagnosed based on ADNI diagnostic criteria and were CSF biomarker positive (Elecsys CSF immunoassay; Aβ_1–42_ < 976.6 pg/ml, p-tau_181_ > 21.8 pg/ml [20]).

### MRI and PET acquisition

All MRI data were obtained on 3-T scanner systems at each ADNI site according to standardized protocol. Tau-PET data were acquired for 30-min dynamic emission scan, six 5-min frames, 75–105 min post-injection of 10.0 mCi of [^18^F]AV1451. Amyloid-PET data were acquired for 20-min dynamic emission scan, four 5-min frames, 50–70 min post-injection of 10.0 mCi of [^18^F]AV45. FDG-PET data were acquired for 30-min dynamic emission scan, six 5-min frames, 30–60 min post-injection of 5.0 mCi of [^18^F]FDG. PET data underwent extensive quality control protocols and standardized image preprocessing correction steps to produce uniform data across the ADNI centers. These steps included frame co-registration, averaging across the dynamic range, and standardization with respect to the orientation, voxel size, and intensity [21]. Detailed information on the imaging protocols and standardized image preprocessing steps for MRI and PET can be found at http://adni.loni.usc.edu/methods.

### MRI and PET preprocessing

T1 MRI images acquired in closest temporal proximity to the tau-PET scan were preprocessed using the same SPM12-based (Wellcome Trust Centre for Neuroimaging, University College London) pipeline as described previously [18]. Briefly, for each subject, the T1 MRI image was segmented into gray matter (GM), white matter (WM), and CSF maps. Next, non-linear high-dimensional spatial normalization parameters were estimated, and a group-specific template was created using SPM’s DARTEL toolbox. The group-specific template was linearly registered to the MNI template in order to estimate the affine transformation parameters.

For each subject, tau-PET, amyloid-PET, and FDG-PET images were coregistered to the participant’s T1 MRI image in native space. For the voxel-based analyses, all PET images were subsequently spatially warped to MNI space using the DARTEL flow fields and affine transformation parameters estimated based on the MRI spatial registration described above. For all PET modalities, standardized uptake value ratio (SUVR) images were computed using the inferior cerebellar gray for tau-PET, the whole cerebellum for amyloid-PET, or the pons for FDG-PET as reference regions. A GM mask was created by warping the group-average GM map from the DARTEL template to MNI space and binarizing the image to only include voxels that had at least 30% GM probability. We further excluded subcortical structures (basal ganglia, thalamus, cerebellum, and brain stem) from the mask because they were either used as reference region or in order to avoid inclusion of regions that show off-target [^18^F]AV1451 binding likely unrelated to tau [22]. All PET images were GM masked and smoothed using an 8-mm Gaussian smoothing kernel.

### Creation of *z*-transformed deviation images (*z*-maps)

To assess differences in tau deposition, we computed voxel-wise mean and standard deviation of SUVR values for CN. The CN group was recruited in ADNI phase III and consisted of 27 amyloid-negative CN subjects with [^18^F]AV1451 tau-PET. *z*-score deviation maps were created for each of the MCI subjects, by subtracting from each voxel the voxel-wise mean and dividing by the standard deviation of CN group SUVR.

### Assessment of amyloid status

Amyloid status was computed using a pre-established protocol [23]. Specifically, T1 MRI images were segmented and parcellated into cortical regions with Freesurfer (v5.3; surfer.nmr.mgh.harvard.edu/), which was used to extract mean amyloid-PET uptake from GM regions (frontal, lateral temporal, lateral parietal, and anterior/posterior cingulate) relative to the whole cerebellum. Participants were classified as amyloid-positive or amyloid-negative based on established cut-points (global amyloid-PET SUVR ≥ 1.11) [23].

### Cognitive assessment

To estimate memory performance, we used ADNI-MEM, an episodic memory composite score based on a broad battery of neuropsychological memory tests [24]. The ADNI-MEM score includes the Rey Auditory Verbal Learning Test, the Alzheimer’s Disease Assessment Scale, the Wechsler Logical Memory I and II, and the word recall of the MMSE.

### Statistical analysis

Demographics were compared between diagnostic groups using *t* tests for continuous variables and chi-squared tests for categorical variables.

We conducted voxel-based linear regression analyses to test the main effect as well as the local interactions amyloid- by tau-PET on FDG-PET. All analyses were controlled for age, gender, education, study site, and - in case of testing the interaction effect - the main effects of amyloid- and tau-PET. All PET measures were included as continuous variables and obtained in spatially corresponding voxels across all three PET modalities, thus assessing the local relationship between the variables. These calculations were done via the software package VoxelStats, a MATLAB (Mathworks Inc., Natick, MA, USA)-based package for multimodal voxel-wise brain image analysis [25]. The customized GM mask (see above) was used to constrain the analysis to cortical GM. The voxel-based statistical parametric maps were corrected for multiple comparisons, where the statistical significance was defined using a random field theory-based [26] threshold of *p* < 0.05 with a cluster forming threshold of *p* < 0.001. In order to examine the nature of the amyloid- by tau-PET interaction, significant voxel clusters of the interactions were identified and labeled according to the largest overlap to the automated anatomical labeling regions. For all three PET modalities, we extracted the mean voxel values within each cluster showing significant amyloid- by tau-PET interactions on FDG-PET resulting from the voxel-wise analyses. We plotted the interactions to ensure that results were not driven by extreme values. The robustness of the interaction effect for each cluster was tested by rerunning the regression model after removing influential cases defined by Cook’s distance *D* [27]. Observations with large influence (the threshold for considering an observation as influential was defined as 4/number of observations) and observations exceeding 3 standard deviations from the mean were excluded in order to test whether the regression coefficient remained significant. Clusters were considered significant and stable when meeting an alpha threshold of 0.05 after removing influential cases.

In addition, post hoc interaction analyses on the mean cluster values were conducted controlling additionally for APOE genotype status (APOE ε4 allele carriers vs non-carriers).

Group-level differences in regional FDG measures were assessed by a one-way ANCOVA (controlling for age, gender, education, and study site) with post hoc *t* test between each pair to assess the difference between MCI subgroups and control groups.

 In order to test whether FDG-PET cluster values were associated with memory performance, we conducted for each cluster a linear regression analysis including ADNI-MEM scores as the dependent variable and the FDG-PET cluster values as the predictor, controlling for age, gender, education, and study site.

All statistical analyses were performed using R-statistical software (http://www.R-project.org). Associations were considered significant when meeting an alpha threshold of 0.05.

## Results

### Sample characteristics

**Fig. 1:**
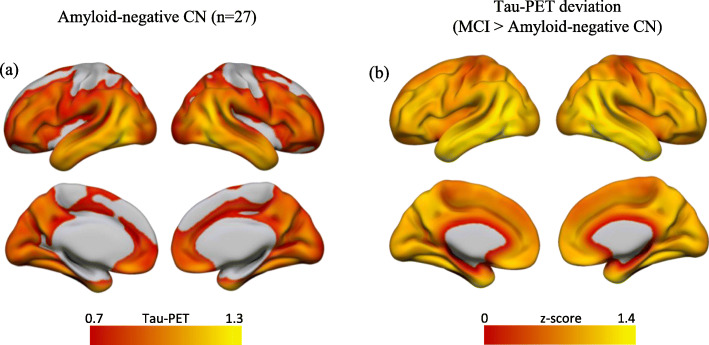
Tau-PET distribution. **a** Mean tau-PET uptake in amyloid-negative CN subjects. **b** *z*-maps of tau-PET deviation in MCI from those in amyloid-negative CN

Demographic characteristics and group differences are presented in Table 1. Figure 1 shows the tau-PET distribution within amyloid-negative CN subjects. Tau-PET levels predominantly in the temporal lobe were higher in MCI compared to those in amyloid-negative CN (Fig. 1b).

**Table 1:** Group characteristics (mean ± SD)

	CN (*n* = 70)	MCI (*n* = 72)	AD dementia (*n* = 95)
Age (years)	72.00 ± 5.48^c^	76.74 ± 7.33	74.11 ± 8.60^a^
Gender (M/F)	33/37	42/30	52/44
Education (years)	16.53 ± 2.65	16.33 ± 2.88	15.48 ± 2.68
MMSE	28.99 ± 1.22^b^	28.22 ± 1.88	22.98 ± 2.04^c^
Aβ−/Aβ+^d^	70/0	42/30	0/95

*Abbreviations*: *Aβ* amyloid-beta, *AD* Alzheimer’s disease, *CN* cognitively normal, *F* female, *M* male, *MCI* mild cognitive impairment, *MMSE* Mini-Mental State Exam

Significantly different from MCI—^a^*p* < 0.05, ^b^*p* < 0.01, and ^c^*p* < 0.001

^d^Aβ status was determined via PET in MCI and via CSF in CN and AD dementia groups

### Voxel-wise amyloid- and tau-PET main effects on FDG-PET metabolism

**Fig. 2:**
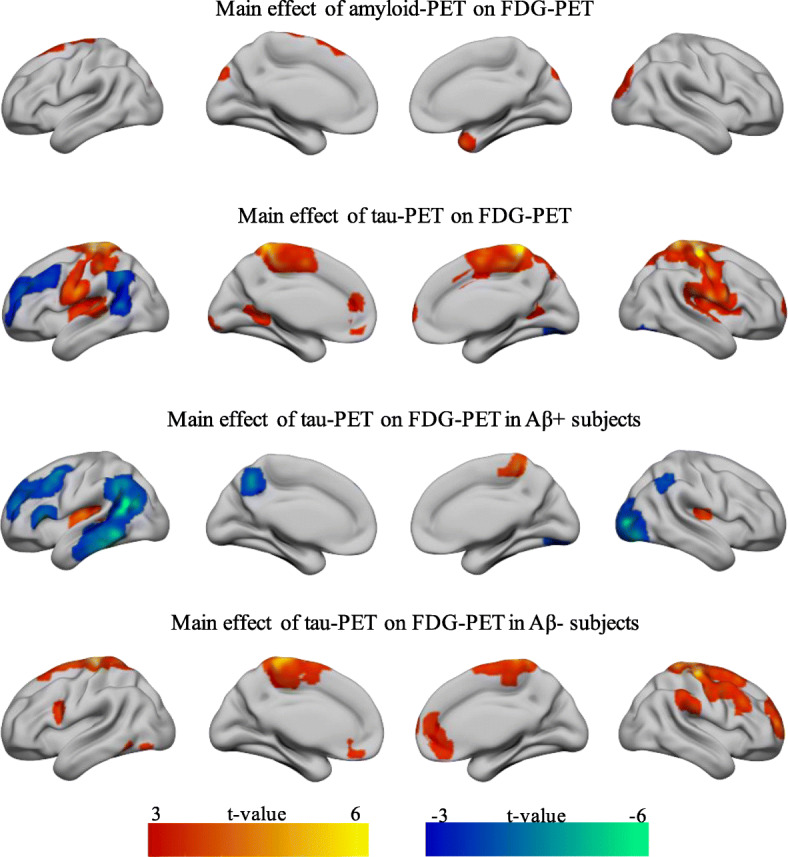
Main effect of amyloid- and tau-PET on FDG-PET metabolism in MCI. Projection of significant clusters resulting from the voxel-wise analysis. MNI coordinates and *t* values of the peaks are provided in supplementary Table 1

First, we tested the main effects of amyloid- and tau-PET on FDG-PET in MCI. As shown in Fig. 2 (for statistics, see supplementary Table 1), higher amyloid-PET was associated with higher FDG-PET in small clusters located in the right superior frontal, right occipital, left cuneus, and right temporal pole. On the other hand, higher tau-PET was associated with higher FDG-PET in multiple regions within the bilateral parietal lobe, left insular, and cingulate cortices. Negative associations were primarily observed within the left middle frontal and left temporoparietal regions.

When stratified by amyloid status (global amyloid-PET SUVR ≥ 1.11), the associations between higher tau-PET and higher FDG-PET metabolism are evident only within the amyloid-negative subgroup, while the opposite association was primarily observed in the amyloid-positive subgroup (Fig. 2, Table 1).

### Voxel-wise amyloid- by tau-PET interactions on FDG-PET metabolism

**Fig. 3:**
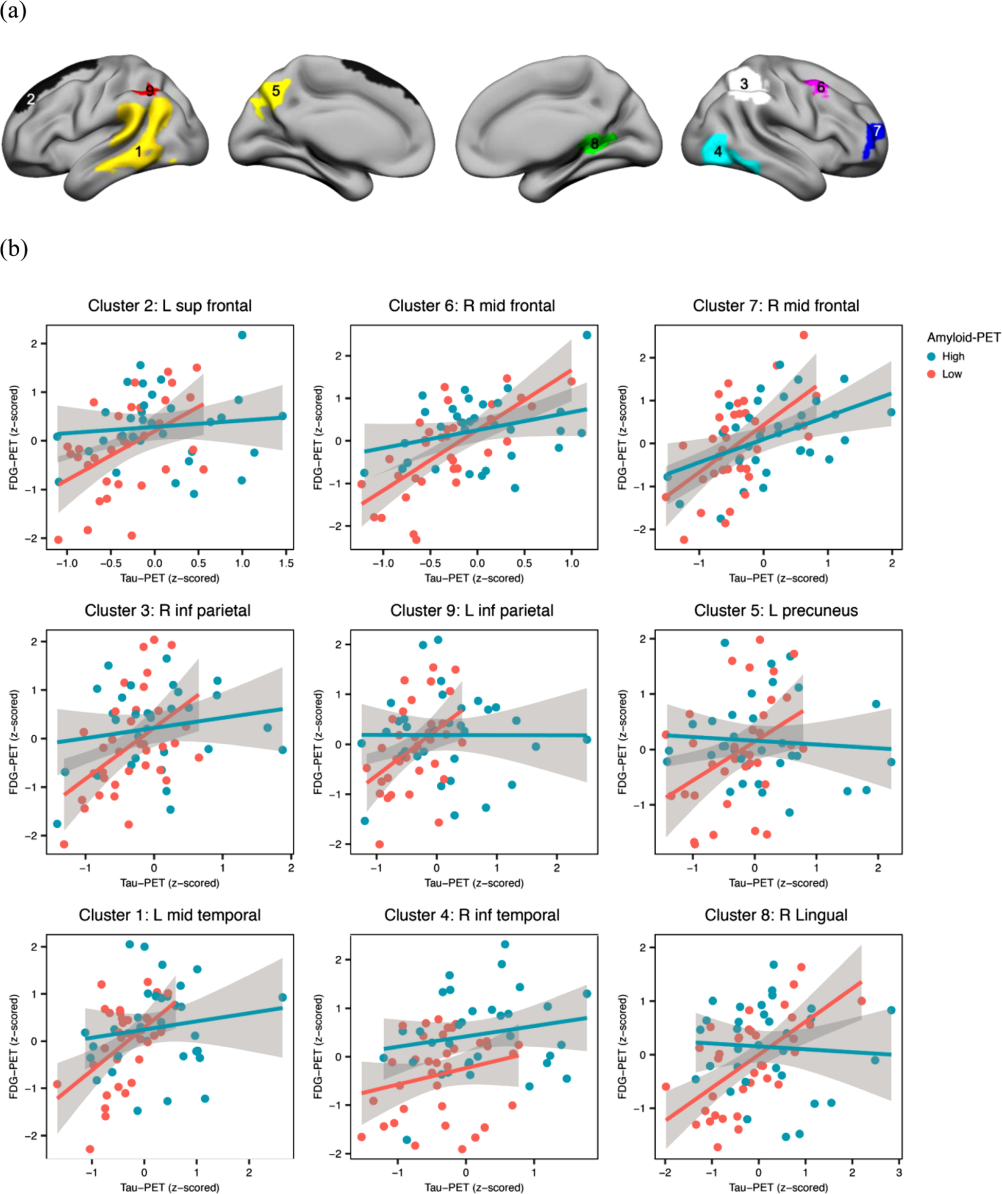
Regional interactions between amyloid- and tau-PET on FDG-PET metabolism in MCI. **a** Projection of significant clusters resulting from the voxel-wise analysis. **b** Scatterplots are based on mean SUVR values extracted from voxel-wise analyses for each of the significant clusters (arranged by anatomical adjacency). For all statistical analyses, amyloid-PET was used as a continuous measure; for illustrational purposes, however, amyloid levels were binarized into high and low levels (median split). Scatterplots are presented after removal of outliers (i.e., defined as influential observations by Cook’s distance and 3 standard deviations from the mean); for regression plots including the outliers, see supplementary Fig. 1

Since we found that the associations between tau-PET and FDG-PET are dependent on Aβ levels, we further tested the local amyloid- by tau-PET interaction on FDG-PET in MCI. Linear regression analysis of the interaction of amyloid-PET by tau-PET (included as continuous variables) showed significant effects in multiple brain regions. In order to examine whether any outliers may drive these interactions, we extracted the mean voxel values in each cluster and examined the undue influence of any observations based on Cook’s distance. Those clusters that survived the quality check are displayed in Fig. 3a (for statistics, see Table 2).

**Table 2:** Areas showing significant voxel-wise interaction between amyloid- and tau-PET on FDG-PET in MCI

Labels	Cluster index	Size (voxels)	***t*** value	MNI coordinates		
				***x***	***Y***	***z***
L middle temporal	1	3552	5.64	− 51	− 60	24
L superior frontal	2	1853	6.4	− 7.5	27	63
R inferior parietal	3	1448	6.11	52.5	− 36	51
R inferior temporal	4	1122	5.29	58.5	− 49.5	− 24
L precuneus	5	606	5.91	− 4.5	− 76.5	39
R middle frontal	6	444	6.24	37.5	10.5	61.5
R middle frontal	7	432	5.07	36	57	13.5
R lingual	8	224	4.39	21	− 48	6
L inferior parietal	9	191	4.25	− 45	− 51	52.5

*L* left, *R* right

MNI coordinates and *t* values of the peaks are provided. *t* values are based on voxel-wise regressions controlling for age, gender, education, and study site.

All amyloid-PET by tau-PET interactions were of the same direction, i.e., higher tau-PET was associated with higher FDG-PET at low levels of amyloid-PET but not at high levels of amyloid-PET (Fig. 3b). These clusters were predominantly located within the left middle temporal gyrus, right inferior temporal gyrus, right lingual gyrus, left precuneus, bilateral inferior parietal gyrus, left superior frontal gyrus, and right middle frontal gyrus.

To determine whether these effects were driven by differences in *APOE* status, we tested whether *APOE* status had influenced the results. When controlling all above listed models for *APOE*, the observed interactions remained significant (*p* < 0.05) in all clusters.

### Tau-related hypermetabolism in amyloid-negative MCI subjects

In order to examine whether the observed tau-related increase in FDG-PET cluster values in the MCI subjects with low amyloid represented abnormal FDG-PET *hyper*metabolism, we compared the FDG-PET cluster values in the MCI subgroups to the FDG-PET in amyloid-negative CN (*n* = 70) and subjects with full-blown AD dementia (*n* = 95). Note that these two reference groups including CN and AD dementia were characterized by CSF biomarker profile of Aβ_1–42_ and p-tau_181_ rather than amyloid- and tau-PET given that those imaging modalities were not available in a sufficiently large number of CN and AD dementia subjects.

**Fig. 4:**
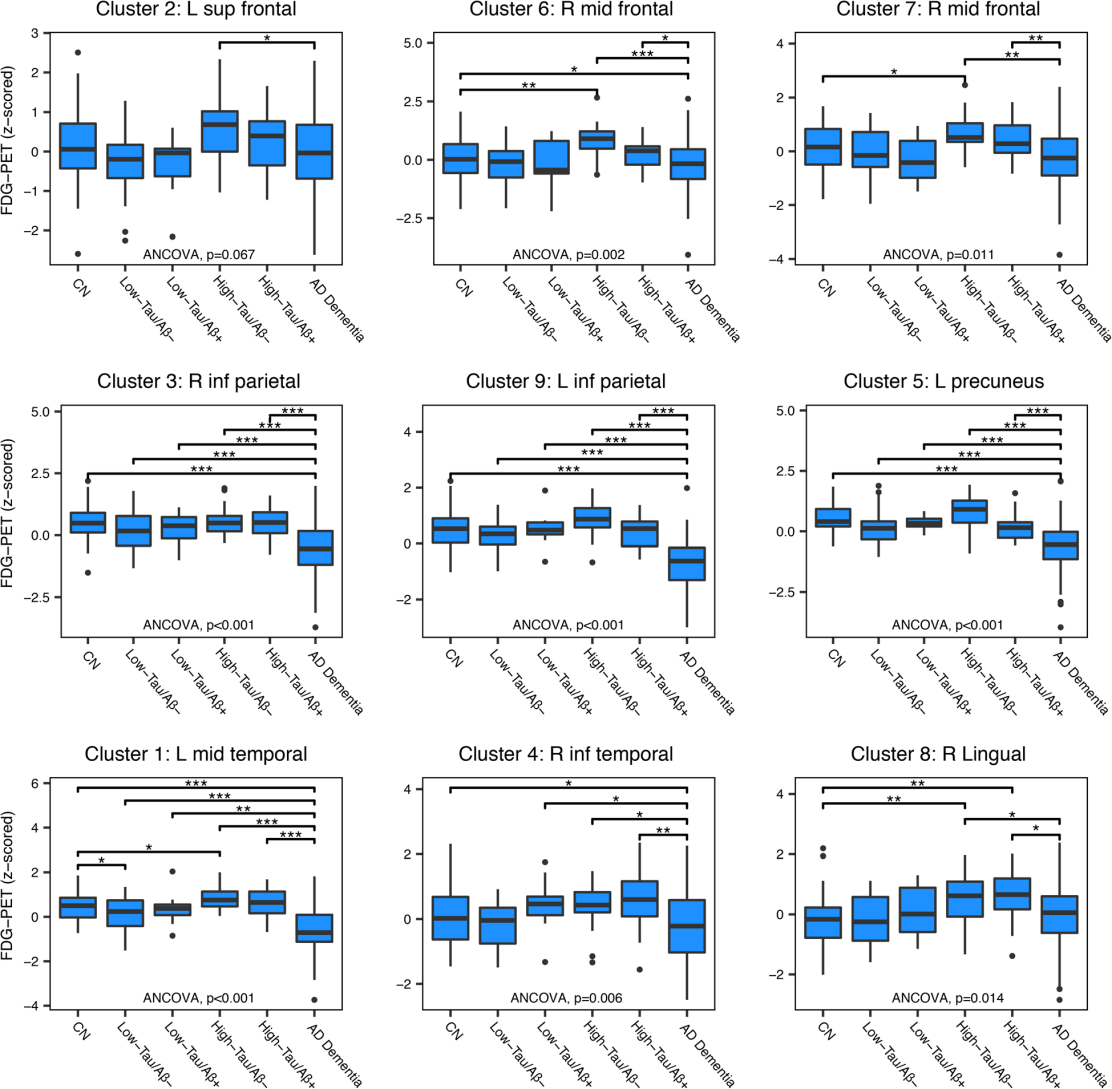
FDG-PET levels in MCI subgroups compared to CN and AD control groups. Mean FDG-PET levels for each cluster (arranged by anatomical adjacency) compared to CN and AD dementia subjects. MCI subjects were stratified by high and low tau PET (median split) and amyloid PET (global amyloid-PET SUVR ≥ 1.11). Significant differences between groups are indicated by *p < 0.05, **p < 0.01, and ***p < 0.001; one-way ANCOVA with post hoc *t* test between each pair

MCI subjects were divided by high and low tau-PET (median split) and by amyloid status (global amyloid-PET SUVR ≥ 1.11), resulting in four subgroups (high vs low tau/positive vs negative amyloid). FDG-PET levels for all MCI subgroups along with the control groups are plotted in Fig. 4. ANCOVA showed significant (*p* < 0.05) group differences in FDG-PET for all clusters except for one cluster within the left superior frontal gyrus (*p* = 0.067). Post hoc analyses confirmed that the tau-related increase in FDG-PET in the high-tau/amyloid-negative MCI subgroup was significantly higher compared to the CN group in clusters located within the right middle frontal, left middle temporal, and right lingual gyri. The same group also had significantly higher FDG-PET levels compared to AD dementia cases within the same clusters, confirming that the FDG-PET levels will eventually decrease with clinical AD progression.

### Hypermetabolism in the right middle frontal cortex is associated with lower memory performance

**Fig. 5:**
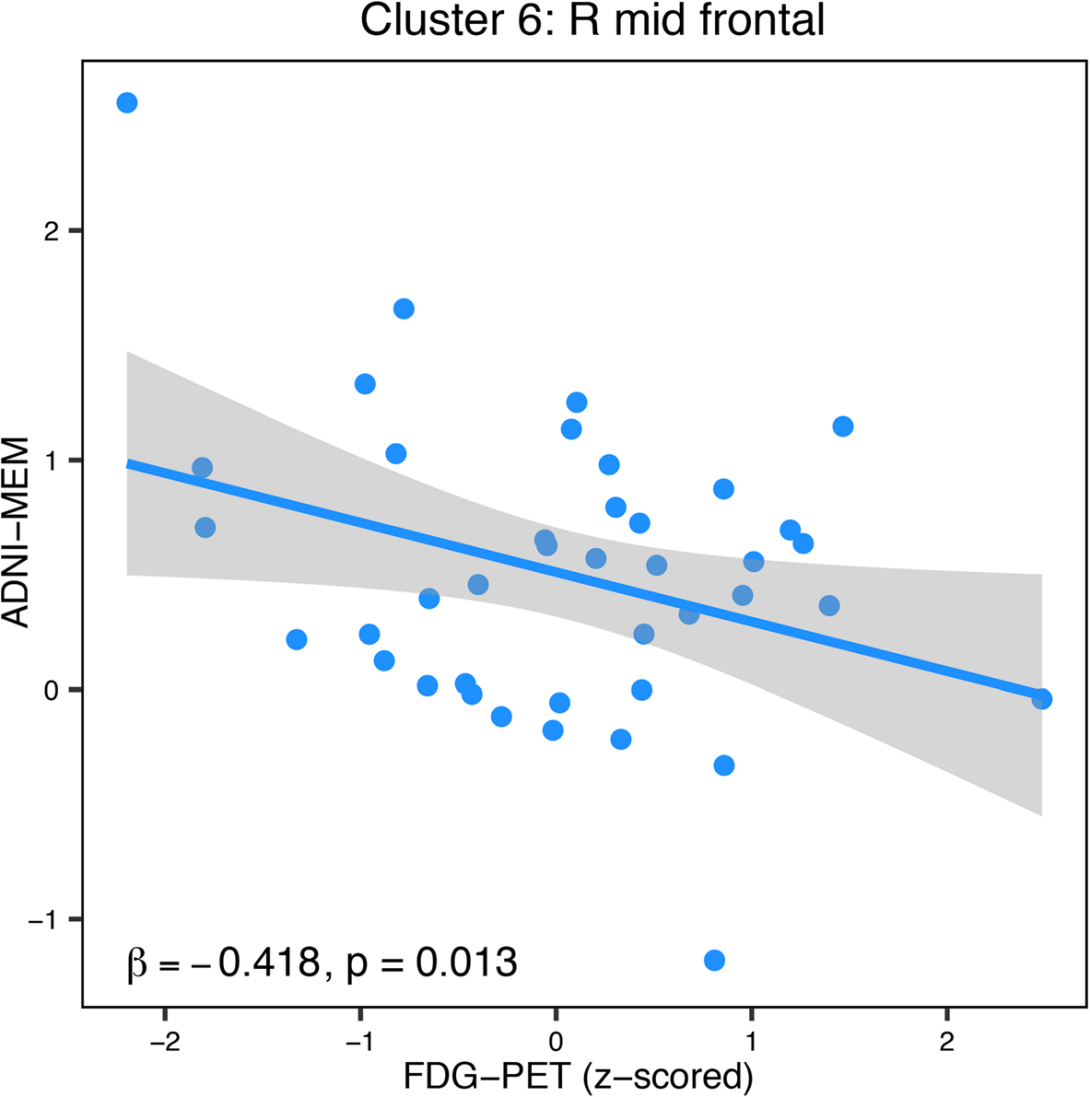
Associations among FDG-PET and memory performance. Scatterplot for the regression model of FDG-PET on ADNI-MEM in amyloid-negative MCI subjects

Next, we addressed the question whether tau-related FDG-PET hypermetabolism in MCI is associated with memory performance. Since FDG-PET hypermetabolism was observed at lower levels of amyloid-PET (see above), we chose to test FDG-PET cluster values as predictors of memory performance in amyloid-negative MCI subjects in each cluster. We found a significant association in the right middle frontal (*p* = 0.013; Fig. 5). The association was negative, meaning higher FDG-PET metabolism in the middle frontal gyrus cluster of FDG-PET hypermetabolism was associated with a lower ADNI-MEM score. This result suggests that right frontal FDG-PET hypermetabolism is associated with worse memory performance. Control analysis in the amyloid-positive MCI subjects did not show significant associations between FDG-PET and cognition for any of the clusters.

## Discussion

Our first major finding showed that higher tau-PET was associated with higher glucose metabolism in subjects with lower levels of amyloid-PET, but not higher levels of amyloid-PET. These effects were predominantly found within the middle temporal gyrus, posterior parietal, and frontal cortex and were independent of APOE genotype. Our second major finding was that the tau-related increases in FDG-PET represented hypermetabolism since the FDG-PET level exceeded that of CN and AD dementia subjects. Our third major finding was that the tau-related FDG-PET hypermetabolism in MCI subjects with low amyloid was associated with lower memory performance.

Our findings advance the current understanding of FDG-PET changes in MCI, providing an explanatory model of FDG-PET hypermetabolism that has been observed in multiple studies in asymptomatic and symptomatic elderly subjects (for a review, see [28]). In line with our results, a recent study in MCI reported increased FDG-PET metabolism at low levels of amyloid-PET but not high levels of amyloid-PET [7]. FDG-PET metabolism was positively associated with Aβ in MCI, but inversely associated with Aβ in AD dementia [29]. We show that tau-PET plays an important role in FDG-PET hypermetabolism in MCI subjects at low Aβ levels, suggesting the interaction of tau and amyloid pathology in non-demented subjects to be key for the increase in FDG-PET. Compared to the interaction approach, our analysis of tau-PET stratified by negative vs positive amyloid-PET showed a more widespread association of higher tau-PET and FDG-PET. Higher tau-PET was preferentially associated with higher FDG-PET in Aβ-negative MCI subjects, but with lower FDG-PET in Aβ-positive subjects, consistent with the results of our interaction analyses. The spatially more restricted interaction effect is probably due to lower statistical power to test an interaction effect compared to testing a main effect.

Our results are consistent with recent findings in CN, where higher tau-PET was associated with higher FDG-PET in participants with low levels of amyloid-PET [15, 16]. We expand significantly above those previous results by showing that the interaction extends to MCI, where the tau-related increase in FDG-PET represents hypermetabolism above normal levels and is associated with lower memory performance. These findings on FDG-PET show parallels to fMRI detected hyperactivation as a function of tau and amyloid pathology. Both resting-state and task-evoked hyperactivity, especially in the medial temporal lobe [30], but also other brain regions [31] has been observed in early-phase autosomal dominant AD [32] and MCI [30, 31, 33]. fMRI-assessed hyperactivation in the medial temporal lobe was associated with faster cognitive decline in MCI [33], consistent with our findings of FDG-PET hypermetabolism to be associated with lower cognitive performance in MCI. Furthermore, fMRI-assessed hyperactivation was associated with higher tau-PET in CN [34, 35]. An interaction of tau-PET by amyloid-PET on resting-state fMRI-assessed network connectivity in CN was observed, such that after a phase of hyperconnectivity, there was a decline in network connectivity when both tau-PET and amyloid-PET were high [36]. These results are reminiscent of the interaction effect of tau-PET by amyloid-PET on FDG-PET observed in the current study. Together, these studies suggest a synergistic interaction of tau and amyloid pathology on brain activity assessed across different modalities.

In the current study, we took a biomarker-centered approach using amyloid- and tau-PET to predict changes in FDG-PET in MCI. A subset of the MCI patients showed no abnormal Aβ levels. Higher tau-PET levels in the absence of abnormal Aβ levels may be due to primary age-related tauopathy (PART) [37]. PART is characterized by elevated tau pathologies confined to Braak-stage regions I–IV at absent or low levels of amyloid plaques and has been proposed to be an etiological entity that is qualitatively different from AD [37, 38]. Although it is still debated whether PART is part of the AD continuum [39], it is generally accepted that abnormal Aβ levels are a defining feature of AD. Thus, not all MCI participants were within the AD continuum. Nevertheless, based on biomarker-driven rather than diagnostic characterization, our study showed that the interaction between both types of AD pathologies is predictive of FDG-PET alterations.

The mechanism by which pathologic tau or amyloid is associated with an increase in glucose metabolism remains an open question. In vitro electrophysiological analysis showed that secreted extracellular tau fragments obtained post-mortem from the brain of an individual with AD cause neuronal hyperactivity in human neurons [40]. Moreover, transgenic mice studies showed that reducing tau protein levels in the brain is associated with reduced susceptibility to neuronal hyperexcitability and seizures [41], suggesting that tau modulates neuronal hyperactivity of neuronal networks [42]. The disruption of GABAergic neuronal network has been suggested as a possible mechanism of tau-associated disturbance of hippocampal neuron excitability [43]. The differential role of tau and amyloid in driving hypermetabolism is somewhat unclear. In transgenic mice expressing amyloid, higher amyloid was linked to higher neural excitability [44]. A recent study in transgenic mouse models of tau and amyloid suggests that amyloid is driving neuronal hyperactivity, but increased levels of tau lead to reduced neuronal activity [45]. However, these results are in conflict with previous results of the amyloid-independent association of tau-related susceptibility to hyperexcitability discussed above [41]. One possibility to reconcile the findings is that tau enhances amyloid-related neuronal hyperactivity at lower levels of amyloid, but reduces neuronal function at higher levels of amyloid. This stance would be in agreement with results from previous studies in humans reporting tau-PET but not amyloid-PET to be linked to fMRI-assessed hyperactivation [35] or FDG-PET hypermetabolism [15, 16]. Furthermore, we observed FDG-PET hypermetabolism in the group of amyloid-negative/high-tau but not amyloid-positive/low-tau suggesting that higher levels of tau in the presence of lower levels of amyloid are decisive for FDG-PET hypermetabolism. As a third alternative, neuronal hyperexcitability may drive initial tau release, propagation, and spread [46, 47]. Future preclinical and intervention studies targeting amyloid or tau pathology will be instrumental in disentangling the causative relationship between primary AD pathologies and FDG-PET hypermetabolism.

Another major finding of our study was the association between FDG-PET hypermetabolism and lower memory performance suggesting that FDG-PET hypermetabolism may reflect pathologically altered FDG-PET levels that are detrimental rather than of compensatory nature. In previous studies including cognitively impaired elderly subjects, increased FDG-PET in the hippocampal formation was associated with poorer cognitive performance [48]. Moreover, reducing hippocampal hyperactivity by drug intervention improves cognition in MCI [49], where the same drug reduced tau-related neuronal hyperexcitability in a transgenic mouse model of AD [50]. Alternatively, higher neural activity may enhance tau spreading which in turn may lead to cognitive decline [46, 47]. To test such a potentially mutually reinforcing chain of events would require longitudinal studies. With the caution that the current study does not allow for a causative interpretation, our findings suggest that local FDG-PET hypermetabolism in the presence of tau has no beneficial effect on cognition. We further caution that the MCI syndrome may have been also caused by other pathologies than amyloid and tau pathologies, especially in the MCI subjects with low amyloid. Alternative pathologies that have been linked to AD-like symptoms include cerebrovascular disease, aggregation of the transactive response DNA binding protein 43 kDa (TDP-43), and alpha-synuclein [51–54].

Several caveats need to be considered when interpreting the results of the current study. First, the current study is cross-sectional in nature. A longitudinal study will be informative to test the predictive value of tau- and amyloid-PET for the subsequent changes in FDG-PET and cognition. Second, the presence of the APOE ε4 allele has been previously shown to be associated with glucose hypermetabolism [6] and thus may provide a confounding variable. However, a post hoc analysis showed that the observed interaction remained significant even when controlling for APOE genotype, suggesting that any association between APOE and tau pathology did not explain the current results. Third, although FDG-PET is commonly interpreted to reflect neural activity, it is possible that FDG-PET also reflects glial activity. For example, microglia activation is increased in relation to tau and amyloid pathology and can be associated with FDG-PET hypermetabolism as suggested by findings in mice [55]. However, our results on FDG-PET show parallels with the findings on resting-state and task-evoked fMRI BOLD signal which is less likely to reflect glia activity, discounting the possibility of glia activation as a major source of PET. Fourth, we did not apply partial volume correction to FDG-PET. We did so deliberately in order to avoid that FDG-PET hypermetabolism may occur due to the correction procedure. Here, we observed increased FDG-PET despite not correcting, supporting the view that a true increase in FDG-PET can be observed as a function of tau and amyloid pathology.

## Conclusions

We found that FDG-PET hypermetabolism occurs as a function of increased tau-PET in the presence of low amyloid-PET, and is associated with worse cognitive performance. Our results have implications for clinical trials, where FDG-PET is often used as an outcome parameter [56]. Given the non-linear changes of FDG-PET as a function of tau and amyloid pathology, a beneficial drug effect on FDG-PET may not always translate into a reduction in the decline of FDG-PET, but could also be a reduction of the detrimental increase in FDG-PET. Clearly, our results call for a more sophisticated model of FDG-PET changes in the course of AD, taking both amyloid- and tau-PET into account.

## Supplementary information

**Additional file 1: Figure S1.** Regional interactions between amyloid- and tau-PET on FDG-PET metabolism in MCI. **Table S1.** Areas showing significant voxel-wise effect of amyloid-PET and tau-PET on FDG-PET in MCI.

## Data Availability

All neuroimaging and neuropsychology data that were used in this study are available online at the ADNI data repository (adni.loni.usc.edu).

## Abbreviations

AD: Alzheimer’s disease
ADNI: Alzheimer’s Disease Neuroimaging Initiative
APOE: Apolipoprotein E
Aβ: Beta-amyloid
CN: Cognitively normal
CSF: Cerebrospinal fluid
FDG: Fluorodeoxyglucose
GM: Gray matter
MCI: Mild cognitive impairment
MMSE: Mini-Mental State Examination
MRI: Magnetic resonance imaging
PET: Positron emission tomography
SUVR: Standard uptake value ratio
WM: White matter
