# Supplementary material for: FDG-PET hypermetabolism is associated with higher tau-PET in mild cognitive impairment at low amyloid-PET levels
Source: Alzheimers Res Ther. 2020 Oct 19;12:133. doi: 10.1186/s13195-020-00702-6 (PMC7574434; doi:10.1186/s13195-020-00702-6)
Supplement: Supplementary file 1 — Additional file 1: Figure S1. Regional interactions between amyloid- and tau-PET on FDG-PET metabolism in MCI. Table S1. Areas showing significant voxel-wise effect of amyloid-PET and tau-PET on FDG-PET in MCI. [file 13195_2020_702_MOESM1_ESM.docx]

**Supplementary**

**(a)**

**
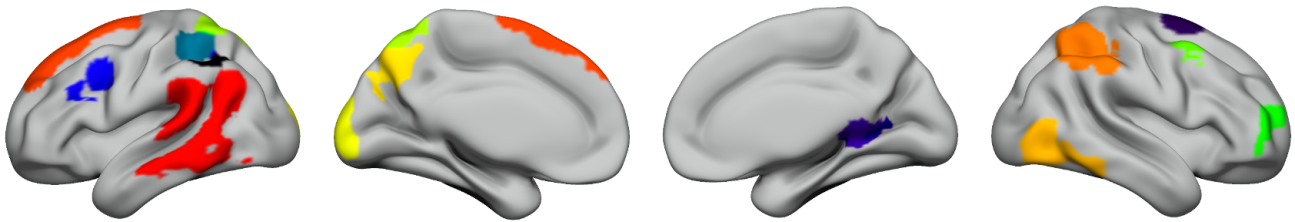
**


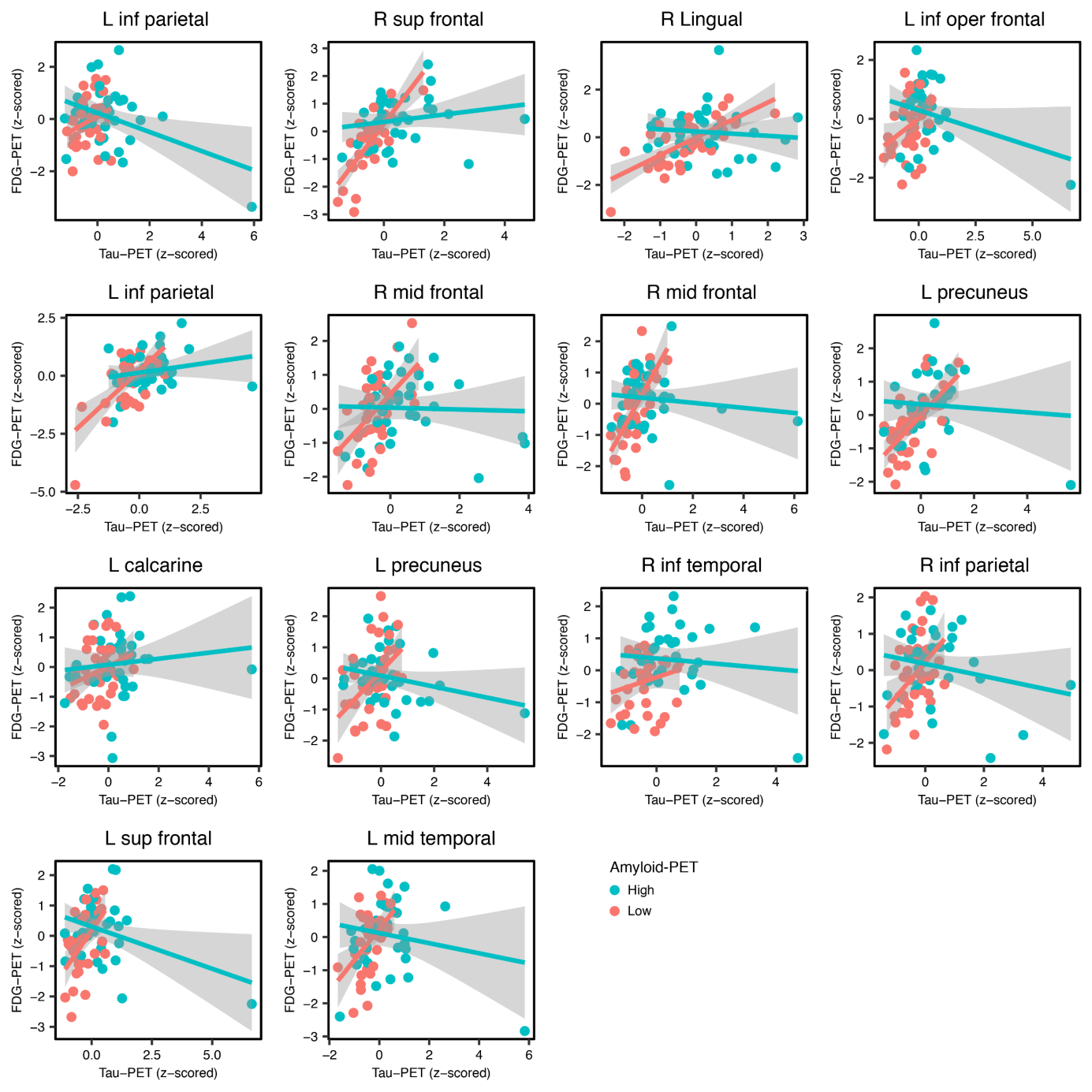
 **Supplementary Figure 1: Regional interactions between amyloid- and tau-PET on FDG-PET metabolism in MCI**. (a) Projection of significant clusters resulting from the voxel-wise analysis. (b) Scatterplots are based on mean SUVR values extracted from voxel-wise analyses for each of the significant clusters (arranged by anatomical adjacency). Amyloid was used as a continues measure, for illustrational purposes amyloid levels were stratified to high and low (median split).

Supplementary Table 1: Areas showing significant voxel-wise effect of amyloid-PET and tau-PET on FDG-PET in MCI.

| **Size**  **(voxels)** | **T-value** | **MNI coordinates** | | |
| --- | --- | --- | --- | --- |
|  |  | **x** | **Y** | **z** |
| Main effect of amyloid-PET on FDG-PET | | | | |
| Positive association | | | | |
| 590 | 4.94 | 24 | 15 | -31.5 |
| 472 | 3.76 | 24 | -90 | 30 |
| 335 | 3.82 | -18 | 12 | 69 |
| 220 | 4.25 | -4.5 | -85.5 | 39 |
| 180 | 4.35 | -13.5 | -12 | 76.5 |
| Main effect of tau-PET on FDG-PET | | | | |
| Positive association | | | | |
| 10844 | 7.55 | 4.5 | -43.5 | 67.5 |
| 4312 | 6.66 | -4.5 | -45 | 70.5 |
| 1439 | 5.05 | -64.5 | -12 | 27 |
| 748 | 5.7 | -49.5 | -24 | 10.5 |
| 410 | 4.28 | -34.5 | -21 | 3 |
| 397 | 4.84 | 15 | 70.5 | 9 |
| 328 | 5.1 | -4.5 | -93 | -18 |
| 244 | 5.81 | 28.5 | -55.5 | 9 |
| 212 | 4.91 | -24 | -54 | 6 |
| 183 | 4.77 | -1.5 | 45 | 15 |
| Negative association | | | | |
| 2124 | 4.94 | -28.5 | 45 | 16.5 |
| 1251 | 4.94 | -49.5 | -52.5 | 9 |
| 564 | 4.62 | 24 | -76.5 | -10.5 |
| Main effect of tau-PET on FDG-PET in Aβ+ subjects | | | | |
| Positive association | | | | |
| 935 | 5.49 | -46.5 | -30 | 9 |
| 280 | 4.67 | 42 | -15 | 15 |
| 241 | 5.34 | 4.5 | -42 | 64.5 |
| Negative association | | | | |
| 5725 | 7.07 | -57 | -48 | -10.5 |
| 2723 | 7.12 | -34.5 | 6 | 51 |
| 1878 | 6.61 | 30 | -90 | 3 |
| 587 | 5.25 | -10.5 | -57 | 46.5 |
| 421 | 5.57 | -48 | 25.5 | 7.5 |
| 322 | 5.2 | 45 | -52.5 | 42 |
| Main effect of tau-PET on FDG-PET in Aβ- subjects | | | | |
| Positive association | | | | |
| 4262 | 5.85 | 36 | -21 | 63 |
| 3091 | 6.96 | -21 | -22.5 | 75 |
| 533 | 4.85 | 27 | 57 | 13.5 |
| 504 | 4.39 | 55.5 | -37.5 | 54 |
| 433 | 4.54 | 3 | 46.5 | 3 |
| 406 | 5.76 | 52.5 | 19.5 | 30 |
| 285 | 4.92 | -19.5 | -48 | 72 |
| 243 | 4.44 | -57 | -63 | -16.5 |
| 220 | 4.67 | -45 | 6 | 16.5 |
| 220 | 4.06 | 21 | 54 | 28.5 |

*L, Left; R, Right.*

*MNI coordinates and t-values of the peaks are provided. T-values are based on voxel-wise regressions controlling for age, gender, education, study site and amyloid/tau-PET.*
